# The gut–brain–circadian axis in anxiety and depression: a critical review

**DOI:** 10.3389/fpsyt.2025.1697200

**Published:** 2025-10-30

**Authors:** Jhommara Bautista, Camila Hidalgo-Tinoco, Miranda Di Capua Delgado, Juliana Viteri-Recalde, Antonio Guerra-Guerrero, Andrés López-Cortés

**Affiliations:** Cancer Research Group (CRG), Faculty of Medicine, Universidad de Las Américas, Quito, Ecuador

**Keywords:** anxiety, depression, gut microbiota, neural pathway, immune pathway, endocrine pathway, metabolic pathway, psychobiotics

## Abstract

Anxiety and depressive disorders rank among the most prevalent psychiatric conditions worldwide, yet remission rates remain unsatisfactory despite advances in pharmacological and psychotherapeutic interventions. The gut–brain axis has emerged as a transformative framework for understanding these disorders, emphasizing bidirectional communication between the central nervous system, the enteric nervous system, the endocrine and immune systems, and the gut microbiota. Preclinical studies demonstrate that germ-free or dysbiotic states exaggerate hypothalamic–pituitary–adrenal (HPA) reactivity, remodel synaptic plasticity, and induce anxiety- and depression-like behaviors, while fecal microbiota transplantation confirms the causal influence of microbial communities. Mechanistically, neural (e.g., vagal), endocrine (e.g., cortisol), immune (e.g., cytokine), and metabolic (e.g., short-chain fatty acids, tryptophan metabolites, bile acids) pathways converge to regulate mood and stress resilience. An underappreciated yet critical dimension of this model is circadian rhythmicity. Both host endocrine cycles and microbial communities exhibit diurnal oscillations that synchronize metabolism, immune activity, and neural signaling. Disruption of these rhythms, through factors such as sleep disturbance, irregular feeding, or shift work, alters microbial diversity, dampens metabolite oscillations, destabilizes HPA regulation, and enhances neuroinflammation, thereby amplifying vulnerability to psychiatric disorders. Collectively, evidence supports a model in which anxiety and depression are systemic conditions arising from integrated neural, immune, endocrine, metabolic, and circadian dysregulation, rather than isolated brain-based pathologies. This reconceptualization positions microbial taxa and metabolites as candidate biomarkers and therapeutic targets. Precision interventions, ranging from diet and psychobiotics to fecal microbiota transplantation, chrononutrition, and immune-modulatory strategies, offer promising avenues for personalized psychiatry.

## Introduction

**Figure 1:**
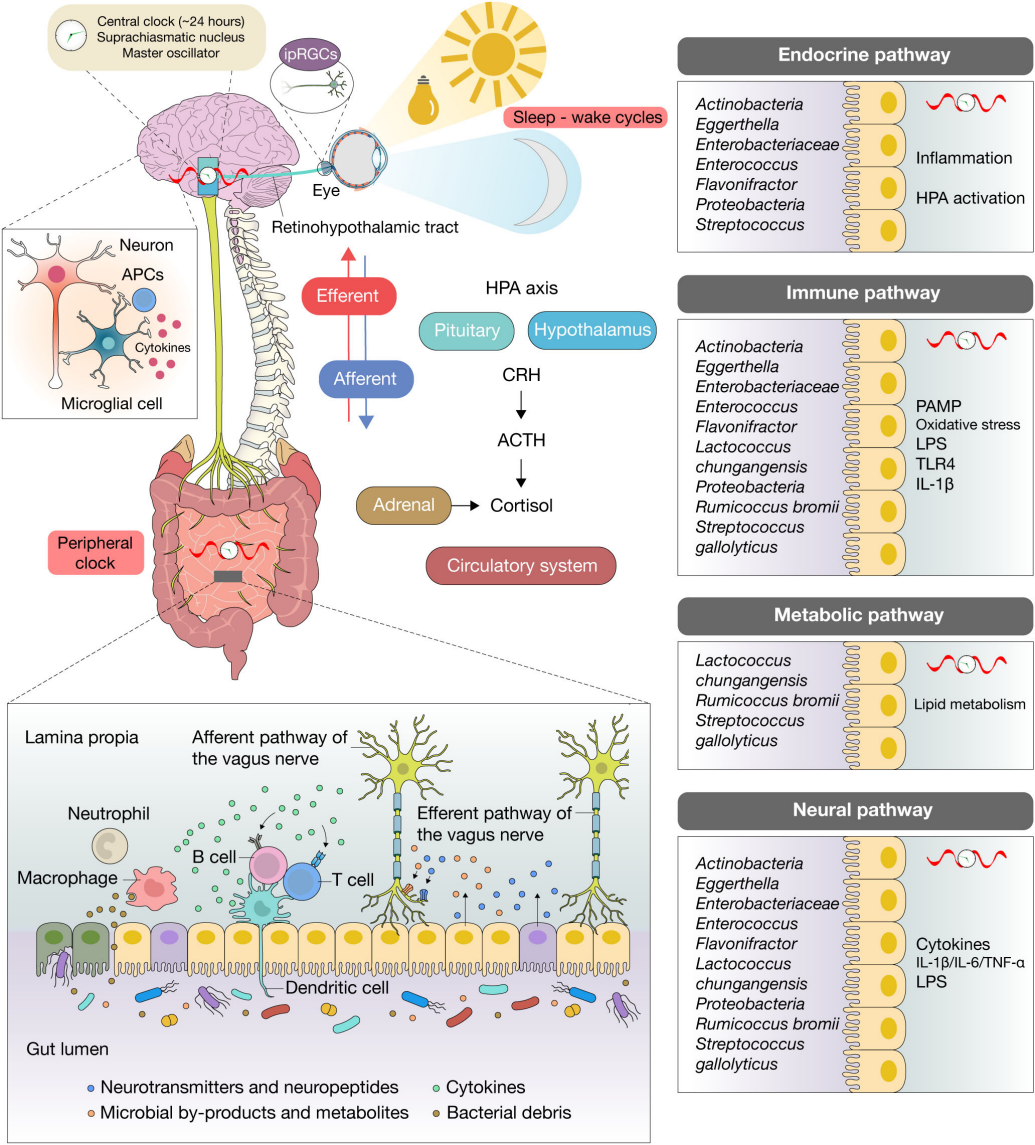
Gut–brain–circadian axis in anxiety and depression. Schematic focusing on risk-enriched microbial signals and their downstream effects across the neural, endocrine (HPA), immune, and metabolic arms of the gut–brain axis: barrier compromise facilitates translocation of PAMPs/LPS, activating TLR pathways and pro-inflammatory cytokines (IL-1β, IL-6, TNF-α) that drive microglial activation, synaptic remodeling, and dysfunction of mood-relevant circuits, contributing to anxiety- and depression-related phenotypes; in parallel, inflammatory signaling diverts tryptophan toward kynurenine via IDO/TDO, diminishing serotonergic tone and generating neuroactive metabolites; stress engages the HPA axis, and cortisol feedback further weakens barrier function and reshapes microbial ecology, creating a feed-forward loop; circadian rhythms and peripheral clocks gate the timing of microbial composition shifts and metabolite fluxes, as well as time-of-day windows for barrier permeability, cytokine responsiveness, vagal tone, and cortisol dynamics; circadian misalignment desynchronizes these rhythms, increases LPS leakage and inflammatory set points, and amplifies risk-associated signaling to the brain.

Anxiety and depressive disorders are among the most prevalent and debilitating psychiatric conditions, collectively affecting nearly 10% of the global population and ranking as leading causes of disability worldwide (1, 2). Despite advances in pharmacological and psychotherapeutic strategies, remission rates remain disappointingly low, underscoring the need for novel mechanistic frameworks to explain disease onset, persistence, and treatment resistance (3). Over the past two decades, the gut–brain axis has emerged as one such paradigm, emphasizing the bidirectional interplay between the central nervous system (CNS), the enteric nervous system (ENS), the endocrine and immune systems, and the intestinal microbiota (4, 5) (**Figure 1**).

The human gastrointestinal tract harbors nearly 10^14^ microbes, outnumbering human cells and carrying a gene pool vastly exceeding the host genome (6–8). These commensals contribute to digestion, immune maturation, and host metabolism, but accumulating evidence reveals their equally critical role in shaping CNS development and behavior (4, 9). Germ-free mice display exaggerated hypothalamic–pituitary–adrenal (HPA) reactivity, anxiety-like phenotypes, and altered synaptic plasticity, which normalize upon colonization with commensals (9, 10). Converging lines of evidence, antibiotic-induced dysbiosis with behavioral rescue after recolonization, monoassociation with specific taxa (e.g., Bifidobacterium infantis) that normalizes HPA output, and metabolite supplementation that restores microglial maturation and synaptic function, support a causal role for microbiota–brain signaling without relying solely on fecal microbiota transplantation (FMT) models (11, 12).

Multiple biological pathways mediate this crosstalk. Neural signaling is exemplified by the vagus nerve, which conveys microbial cues to mood-related brain circuits; indeed, vagotomy abolishes the behavioral effects of probiotics and FMT (6, 13). Endocrine routes converge on the HPA axis, where dysbiosis disrupts glucocorticoid rhythms, elevates cortisol, and exacerbates stress sensitivity (3, 14). Immune signaling provides another conduit: lipopolysaccharide (LPS) and other pathogen-associated molecular patterns can breach a compromised gut barrier, triggering cytokine release such as IL-1β, IL-6, and TNF-α, which activate microglia and remodel synapses (9, 15, 16). Metabolic signaling represents a further layer of complexity, as microbiota-derived short-chain fatty acids (SCFAs) regulate blood–brain barrier (BBB) integrity, synaptic plasticity, and anti-inflammatory tone, while tryptophan metabolites influence serotonin biosynthesis and kynurenine pathway flux (11, 17, 18). Together, these pathways establish a framework in which microbial imbalance, or dysbiosis, contributes to psychiatric vulnerability.

Importantly, SCFAs and tryptophan metabolites exert convergent but mechanistically distinct influences on the CNS. SCFAs primarily act *via* G-protein–coupled receptors (FFAR2/FFAR3) and histone deacetylase inhibition, modulating microglial maturation, neurotransmission, and neuroinflammation (19). In parallel, tryptophan-derived metabolites access the brain through LAT1 transporters and act *via* serotonin receptors, kynurenine–N-methyl-D-aspartate (NMDA) signaling, and the aryl hydrocarbon receptor (AhR), ultimately producing overlapping outcomes such as modulation of mood, synaptic function, and stress reactivity (20–22). Alterations in bile acid pools further modulate dopaminergic and noradrenergic circuits, affecting limbic regions such as the prefrontal cortex and amygdala that are critical for mood regulation (23). Concurrently, these metabolic shifts enhance systemic inflammation by amplifying IL-6 and TNF-α release, priming microglia, and compromising the BBB integrity, mechanisms long implicated in depression and other affective disorders (24). Collectively, these findings establish a mechanistic link between microbial dysbiosis, aberrant neurotransmission, and neuroimmune dysfunction.

Historically, depression was viewed primarily through neurochemical models, but immunological perspectives now dominate (25). Peripheral immune activation induces “sickness behavior,” an adaptive, cytokine-driven syndrome characterized by anhedonia, fatigue, and social withdrawal, that overlaps strikingly with core features of depression (15, 26). Proinflammatory cytokines cross the BBB and modulate neurotransmitter systems including serotonin, dopamine, and glutamate, thereby precipitating behavioral and cognitive changes (3, 24). Evolutionary theories posit that depressive behaviors, while maladaptive in modern societies, once conferred survival benefits by conserving energy during infection or injury (3). Contemporary studies confirm that individuals with heightened inflammatory responses to psychosocial stressors are at greater risk of developing depression, reinforcing the gut–immune–brain axis as a central mechanism (9, 27).

Human studies now provide strong evidence for these links. Large-scale metagenomic analyses, such as those from the Flemish Gut Flora Project, revealed that butyrate-producing genera like *Faecalibacterium* and *Coprococcus* are associated with improved quality of life, whereas their depletion, along with reduced *Dialister*, correlates with depression (11). In contrast, enrichment of pro-inflammatory taxa such as *Eggerthella* and *Enterobacteriaceae* has been reported in MDD patients. Multi-omics studies consistently demonstrate that these microbial signatures are mirrored by metabolic shifts, reduced SCFAs, indoles, and serotonin precursors alongside elevated kynurenine pathway metabolites, that converge to impair neurotransmitter balance and promote chronic neuroinflammation (11, 18, 28, 29). These microbial signatures are paralleled by metabolic shifts, including reduced SCFAs and indoles and increased kynurenine pathway metabolites, all of which influence neurotransmitter signaling and immune activation (4, 11). Clinically, many patients with depression and anxiety also present with gastrointestinal comorbidities, highlighting the translational relevance of the microbiota–gut–brain axis (4).

An often-overlooked dimension in this model is the role of circadian rhythms. Both host endocrine cycles, such as cortisol and melatonin secretion, and microbial communities display diurnal oscillations that synchronize metabolic and neural processes (9, 30). Disruption of these rhythms through factors such as shift work, irregular feeding, or sleep disturbances alters microbial diversity, dampens SCFA oscillations, and destabilizes HPA regulation (4, 31). Experimental evidence now shows that circadian rhythm disruption (CRD) induces disturbed expression of the intestinal epithelial gene *Per2*, drives gut microbiota dysbiosis, and *via* FMT, transfers depression-like phenotypes to naïve recipients, thereby implicating microbiota circadian misalignment as a causal driver of mood vulnerability (9, 32, 33). Recent studies further reveal that circadian disruption not only exacerbates neurotransmitter dysregulation but also heightens systemic inflammation, thereby creating a temporal dimension to gut–brain–immune interactions (31, 34, 35). These findings integrate circadian biology into the gut–brain–immune framework and open the door to chronotherapeutic and microbiota-targeted interventions.

Together, this evidence converges on the conclusion that microbial dysbiosis contributes causally to the onset and persistence of anxiety and depression (6, 29). This reconceptualizes psychiatric disorders as systemic conditions arising from the intersection of neural, endocrine, immune, metabolic, and circadian networks, all shaped by the microbiota, rather than isolated brain-based pathologies. Beyond mechanistic insights, these findings have translational implications: microbial taxa and metabolites are emerging as biomarkers for disease risk and treatment response, while interventions such as psychobiotics, FMT, precision diets, chrononutrition, and immune-modulatory therapies hold promise for individualized psychiatric care (9, 36).

## The gut–brain axis in the pathophysiology of anxiety and depression

The gut–brain axis has emerged as a central conceptual framework for understanding anxiety and depressive disorders, emphasizing the bidirectional communication between the CNS, the ENS, the endocrine and immune systems, and the gut microbiota (6, 37, 38). Communication across this axis occurs through neural, endocrine, immune, metabolic, and circadian pathways that collectively orchestrate stress responses, emotional regulation, and systemic homeostasis. Neural signaling is exemplified by the vagus nerve, which transmits microbial cues directly from the gut to the brain; indeed, vagotomy abolishes depression-like behaviors induced by FMT from depressed individuals into rodents, demonstrating a causal link between microbial signals and mood regulation (39, 40). Endocrine pathways converge on the HPA axis, where microbial alterations influence glucocorticoid rhythms, cortisol secretion, and stress reactivity (41–43). Immune mechanisms provide another conduit: LPS and other microbial components can cross a compromised gut barrier, triggering systemic cytokine release such as IL-1β, IL-6, and TNF-α, which activate microglia, remodel synaptic function, and promote neuroinflammation (19, 41). In parallel, microbial metabolites such as SCFAs enhance BBB integrity and synaptic plasticity, while tryptophan metabolites regulate serotonin and kynurenine pathways that influence mood and cognition (6, 23). Importantly, these neural, endocrine, immune, and metabolic pathways are not temporally static, they are entrained by circadian clocks, and disruption of daily rhythmicity amplifies gut dysbiosis, HPA hyperactivity, and neuroinflammatory tone, ultimately heightening vulnerability to anxiety and depression (31, 44–47) (**Table 1**).

**Table 1:** Integrated gut–brain–circadian axis in anxiety and depression: mechanisms, microbial signatures, and therapeutic opportunities.

Axis (primary pathway)	Key mechanistic nodes	Microbial signatures (taxa/metabolites)	Clinically useful biomarkers	Dominant phenotypes linked to anxiety/depression	Therapeutic strategies	Chronotherapy and timing guidance	Evidence level*
Neural (Vagal and Autonomic; Neurotransmitters)	Vagal afferents connecting ENS with limbic and prefrontal circuits; modulation of synaptic plasticity; regulation of GABA, serotonin, dopamine, norepinephrine, and glutamate systems	Altered abundance of Faecalibacterium, Coprococcus, Bifidobacterium, Lactobacillus, Lactococcus chungangensis, Streptococcus gallolyticus, and Ruminococcus bromii; increased Proteobacteria and Eggerthella; altered SCFAs, indoles, and kynurenine metabolites	Fecal SCFAs (low butyrate); plasma kynurenine / tryptophan ratio; cortisol AUC; fronto-limbic reactivity on imaging	Hyperarousal, anhedonia, gastrointestinal comorbidity, impaired stress coping	Psychobiotics (B. longum, L. helveticus, L. gasseri), prebiotics, diet enrichment for butyrate producers, physical exercise, vagal-tone training	Psychobiotics administered in the morning or early afternoon; stable meal times; avoid late-night feeding	Preclinical + Clinical
Endocrine (HPA Axis)	CRH–ACTH–cortisol signaling, glucocorticoid receptor sensitivity, hippocampal BDNF regulation, developmental programming of stress circuits	Dysbiosis with reduced SCFA producers and increased LPS load; Mycobacterium vaccae promoting regulatory immunity	Flattened diurnal salivary cortisol rhythm, cortisol awakening response, hair cortisol, serum BDNF	Stress hypersensitivity, cognitive dysfunction, sleep disturbance	Omega-3 PUFAs, vitamin A, psychobiotics reducing cortisol, stress-reduction and mindfulness programs	Early-day feeding (08:00–16:00) to restore cortisol rhythm; bright-light exposure after waking	Preclinical + Clinical
Immune (Peripheral-to-CNS; Microglia and BBB)	Activation of TLR and NLR pathways, NLRP3 inflammasome, cytokines IL-1β, IL-6, TNF-α, microglial priming, blood–brain-barrier integrity (claudin-5, occludin)	Increased Proteobacteria and Streptococcus gallolyticus (pro-inflammatory signature in MDD cohorts); loss of butyrate producers; decreased indoles and SCFAs; altered bile-acid ratios	hs-CRP, IL-6, TNF-α, LBP, sICAM-1, sVCAM-1, markers of BBB leakage; TSPO PET imaging	Fatigue, psychomotor slowing, anergia, cognitive fog	Anti-inflammatory adjuncts in inflamed subtypes (e.g., TNF-α inhibitors), polyphenol-rich diet, fibers enhancing butyrate and barrier function	Anti-inflammatory treatments scheduled in the morning; consistent meal and sleep times	Preclinical + Stratified Clinical
Metabolic (Microbial Mediators)	SCFAs acting through FFAR2/3 and HDAC inhibition; tryptophan metabolism via IDO/TDO pathways; indoles activating AhR; bile-acid signaling through FXR/TGR5	Decreased SCFA producers (Ruminococcus bromii, Faecalibacterium prausnitzii); decreased indoles; increased quinolinic acid; altered lithocholic and deoxycholic acids; lipidomic remodeling	Fecal butyrate and propionate; serum indole derivatives; kynurenine / tryptophan ratio; bile-acid panels	Low mood, excitotoxic drive, metabolic and visceral dysregulation	Mediterranean-style diet, targeted prebiotics (inulin, GOS), postbiotics (butyrate), bile-acid modulators (under study)	Time-restricted eating to restore rhythmic SCFA and indole oscillations; avoid late eating	Preclinical + Clinical / Observational
Circadian (Host–Microbiome Synchrony)	Core clock genes (CLOCK, BMAL1, PER, CRY), SCN–peripheral communication, microbial diurnal oscillations entrained by feeding, melatonin–cortisol coupling	Flattened microbial and metabolite rhythms; disrupted oscillations of SCFAs and indoles; intestinal Per2 disturbances	Actigraphy, sleep logs, DLMO, melatonin and cortisol rhythmicity, clock-gene expression	Mood instability, insomnia, poor stress resilience	Chrononutrition, early time-restricted eating, morning bright-light therapy, regular sleep–wake cycles, chrono-probiotics (L. gasseri CP2305)	Bright-light therapy 30–60 min in morning; early feeding window; probiotics and polyphenols administered in morning; avoid evening stimulants	Preclinical + Meta-Analyses / Early Clinical
Cross-Axis Integration (Systems View)	Coupling among neural, endocrine, immune, and metabolic pathways; ENS, vagal, HPA, and microglial interactions; barrier integrity; life-stage sensitivity windows	High quality-of-life correlates with Faecalibacterium and Coprococcus; MDD associated with Eggerthella and Enterobacteriaceae	Composite biomarker panels (low fecal butyrate plus high Kyn / Trp and elevated hs-CRP plus gastrointestinal burden)	Distinct subtypes including inflamed, circadian-misaligned, and metabolically rigid patterns underlying treatment resistance	Stepped-care model encompassing diet, psychobiotics, chronobehavioral therapy, FMT, and biomarker-guided anti-inflammatory agents	Diagnostic and therapeutic actions scheduled according to biological clock (morning sampling and dosing)	Integrative framework supported across domains

ACTH, adrenocorticotropic hormone; AhR, aryl hydrocarbon receptor; BBB, blood–brain barrier; BDNF, brain-derived neurotrophic factor; CRH, corticotropin-releasing hormone; DLMO, dim-light melatonin onset; ENS, enteric nervous system; FFAR2/3, free fatty acid receptors 2 and 3; FMT, fecal microbiota transplantation; FXR, farnesoid X receptor; GABA, gamma-aminobutyric acid; GOS, galacto-oligosaccharides; HDAC, histone deacetylase; HPA, hypothalamic–pituitary–adrenal; hs-CRP, high-sensitivity C-reactive protein; IDO/TDO, indoleamine/tryptophan 2,3-dioxygenase; IL, interleukin; LBP, lipopolysaccharide-binding protein; LPS, lipopolysaccharide; MDD, major depressive disorder; NLR, nucleotide-binding oligomerization domain-like receptor; NLRP3, NLR family pyrin domain–containing 3; NMDA, N-methyl-D-aspartate receptor; PUFA, polyunsaturated fatty acid; RCT, randomized controlled trial; SCFA, short-chain fatty acid; SCN, suprachiasmatic nucleus; sICAM-1, soluble intercellular adhesion molecule-1; sVCAM-1, soluble vascular cell adhesion molecule-1; TLR, toll-like receptor; TNF-α, tumor necrosis factor alpha; Trp, tryptophan; TSPO, translocator protein; TGR5, G-protein-coupled bile acid receptor 1.

Preclinical studies have established that the microbiota is an active regulator of stress circuitry rather than a passive correlate. Germ-free mice exhibit exaggerated HPA responses and anxiety-like behaviors that normalize following colonization with commensal microbiota, demonstrating that microbial input is essential for appropriate stress regulation (6, 37, 48). Complementary strategies, perturbation, replacement, and rescue, further reinforce causality: antibiotic-induced dysbiosis elicits anxiety- and depression-like phenotypes reversible by recolonization; monoassociation with *Bifidobacterium infantis* or selected *Lactobacillus* species recalibrates HPA tone and reduces behavioral despair; supplementation with microbial metabolites such as butyrate or indole derivatives restores microglial maturation, BBB integrity, and synaptic plasticity; and vagal deafferentation abolishes probiotic-specific behavioral effects, confirming that neural pathways mediate microbial influence on mood (39, 49). These converging lines of evidence highlight that gut microbial signals actively shape affect-related neural circuitry through endocrine and immune cross-talk.

Beyond these mechanisms, emerging data reveal that the gut microbiota and the host circadian system are dynamically intertwined. The microbiota exhibits robust diurnal oscillations in composition, localization, and metabolic activity that align with host feeding–fasting cycles and hormonal rhythms. These microbial rhythms, in turn, entrain peripheral clocks within intestinal, hepatic, and immune tissues, coordinating metabolic and neuroendocrine homeostasis (31, 46). Microbial depletion through antibiotics disrupts rhythmic gene expression in the hippocampus, amygdala, and SCN, alters corticosterone rhythmicity, and produces time-of-day–dependent changes in stress reactivity, indicating that microbial cues synchronize neuroendocrine rhythms (50). Transcriptomic analyses further show enrichment of dysregulated genes related to BBB permeability, circadian timing, and stress adaptation, revealing a molecular bridge between microbial signals and host clock machinery (50). Likewise, gut microbes drive daily oscillations in tryptophan metabolism: microbial regulation is required for the diurnal production of serotonin and kynurenine metabolites that modulate mood-related neurotransmission (51). Conversely, chronic stress flattens microbial rhythmicity; in depression-model mice, restraint stress abolishes circadian oscillations of key microbial taxa and metabolic pathways (52). Loss of host clock genes such as *Clock* or *Bmal1* produces parallel effects, erasing microbial oscillations and disrupting metabolic synchrony (31).

At the systems level, meal timing emerges as a dominant synchronizer of microbiota–clock interactions. Irregular feeding schedules blunt microbial oscillations and propagate circadian misalignment across the gut–brain–immune axis, leading to metabolic, inflammatory, and behavioral dysregulation (53). Together, these findings indicate that gut dysbiosis in mood disorders reflects not only compositional imbalance but also a loss of temporal order, flattened microbial rhythms, mistimed metabolite release, and desynchronized clocks across host tissues. This concept redefines dysbiosis as a dynamic, time-sensitive disruption of microbiota–host synchrony rather than a static state of imbalance.

Clinical studies reinforce these mechanistic insights. Large-scale metagenomic analyses in major depressive disorder (MDD) reveal depletion of butyrate-producing genera such as *Faecalibacterium prausnitzii* and *Coprococcus*, alongside enrichment of proinflammatory taxa including *Enterobacteriaceae* (41, 54, 55). These microbial shifts correlate with systemic inflammation, impaired intestinal barrier integrity, and altered connectivity in emotion-related brain regions (42, 56). Moreover, microbial and metabolite signatures correlate with treatment response, suggesting their potential as biomarkers to stratify patients and predict therapeutic outcomes (54, 56). When integrated with circadian evidence, these findings suggest that psychiatric vulnerability arises from temporal desynchrony across microbial, immune, and neural systems, and that restoring rhythmic harmony through interventions such as time-restricted feeding, chrononutrition, or circadian-timed psychobiotics may enhance therapeutic efficacy.

Collectively, evidence from animal models and human cohorts supports a model in which gut microbial dysbiosis contributes causally to the onset and persistence of anxiety and depression through integrated neural, endocrine, immune, metabolic, and circadian pathways. This time-sensitive systems framework redefines psychiatric disorders as dynamic, body-wide conditions shaped by microbiota–clock co-regulation. It also underscores the need for chronobiologically optimized interventions, ranging from diet and microbiome modulation to light–sleep alignment, to re-entrain disrupted microbial and host rhythms for improved mental health outcomes.

## Neural circuits and neurotransmitter modulation in the gut–brain axis

The microbiota shapes anxiety- and depression–relevant emotional and cognitive functions through well-defined neuroanatomical circuits that connect the gut with the CNS (57). The vagus nerve represents the most prominent communication pathway, transmitting microbial and luminal signals from the gastrointestinal tract to brainstem nuclei implicated in mood regulation and projecting to limbic/prefrontal targets that govern fear, negative affect, and stress coping (58). Experimental studies show that probiotics alter vagal afferent activity, while vagotomy abolishes these effects, eliminating anxiolytic/antidepressant-like behavioral responses and confirming vagal dependency (6, 59). Complementing this route, spinal and sympathetic pathways convey microbial cues related to visceral nociception, stress responsiveness, and autonomic regulation which can amplify hyperarousal and stress reactivity typical of anxiety and depressive phenotypes (4, 60). Within the ENS, microbial metabolites modulate neuronal excitability and gut motility, reshaping interoceptive signaling that feeds forward to affective circuits and helps explain frequent GI comorbidity in anxiety and depression (61, 62). In what follows, we focus specifically on how the microbiota modulates core neurotransmitter systems that mechanistically link gut signals to anxiety and depressive symptom domains.

Beyond structural connectivity, the microbiota exerts profound effects on neurotransmitter systems. Several bacterial taxa directly synthesize or regulate bioactive amines. *Lactobacillus* and *Bifidobacterium* species produce γ-aminobutyric acid (GABA), a key inhibitory neurotransmitter strongly linked to anxiety phenotypes (60, 63, 64). Reduced abundance of GABA-producing taxa is associated with heightened amygdala reactivity and anxiety-like behavior, whereas probiotic supplementation that increases microbial GABA production correlates with decreased behavioral despair and reduced physiological stress markers in preclinical and clinical settings (60, 63, 64). Microbiota also influence serotonergic tone through interactions with enterochromaffin cells: spore-forming bacteria stimulate peripheral serotonin biosynthesis, which constitutes a major portion of circulating serotonin that regulates both gut motility and central serotonergic signaling (6, 9). In depressive phenotypes, dysbiosis diverts tryptophan away from 5-HT synthesis toward the kynurenine pathway, contributing to anhedonia and low mood; restoration of eubiotic communities or targeted psychobiotics increases 5-HT–related signaling and tracks with symptom improvement (6, 9, 65). Dopaminergic and noradrenergic systems are similarly modulated by microbiota-derived metabolites, including SCFAs and tryptophan catabolites, which alter catecholamine synthesis and receptor sensitivity in mood-related brain regions such as the prefrontal cortex and amygdala (7, 28, 61). These catecholaminergic effects map onto motivational deficits, psychomotor slowing, and heightened arousal commonly observed in depression and anxiety, linking microbial metabolite availability to reward processing and stress responsivity (66, 67).

Microbial regulation of neurotransmission extends beyond production to the modulation of synaptic plasticity and receptor signaling. Indole derivatives and bile acid metabolites cross the intestinal barrier and act on neuronal and glial targets, reshaping synaptic connectivity in cortical and limbic regions (60, 62). Indole–AhR signaling supports serotonergic and glutamatergic balance and dampens microglial activation, thereby constraining anxiety-like behavior and depressive affect; loss of indole producers removes this brake on excitatory tone and neuroinflammation (68, 69). SCFAs also play a pivotal role in restoring microglial maturation and dampening neuroinflammation in germ-free mice, suggesting that microbial metabolites not only influence neurotransmitter availability but also support cellular processes fundamental for CNS homeostasis (9, 17). Functionally, butyrate’s histone deacetylase (HDAC) inhibition enhances expression of GABAergic and neurotrophic genes, normalizes synaptic plasticity in stress-responsive circuits, and is repeatedly linked to reductions in anxiety- and depression-like behaviors (70).

Glutamatergic signaling provides a key convergence point for anxiety and depression. Microbiota-driven activation of indoleamine 2,3-dioxygenase (IDO) shifts tryptophan toward kynurenine metabolites; quinolinic acid (NMDA agonist) promotes excitotoxicity and anxiety/depressive behaviors, while kynurenic acid (NMDA antagonist) can be neuroprotective (71–74). Elevated Kyn/Trp ratios in dysbiosis thus index a neurotransmitter imbalance (low 5-HT, high Glu/NMDA) that correlates with symptom severity and treatment resistance. Interventions that restore SCFAs and indoles, or reduce peripheral inflammation (which drives IDO), rebalance this axis and improve affective outcomes (75, 76).

Neurotransmitter dynamics are further synchronized by circadian clocks, which regulate their synthesis and release (34). Microbial metabolites such as SCFAs and indoles exhibit daily oscillations that tune GABAergic, serotonergic, and dopaminergic signaling. Disruption of these rhythms compromises stress resilience and mood stability, providing an additional temporal dimension to gut–brain interactions (44, 77, 78). Accordingly, timing interventions (chrononutrition, circadian-aligned psychobiotics) to coincide with peak microbial metabolite release enhances GABAergic tone and stabilizes monoaminergic balance, offering a mechanistic rationale for time-of-day–specific treatment windows in anxiety and depression (35, 79–81).

Importantly, the relationship between neurotransmission and microbiota is reciprocal. Stress and altered neural activity reshape gut microbial composition, which in turn modifies neurotransmitter availability and signaling efficacy (4, 6). Chronic stress reduces microbial diversity and disrupts metabolic outputs such as SCFAs and indoles, thereby reinforcing maladaptive neurotransmission patterns underlying anxiety and depression (7, 82). This creates a feed-forward loop, stress hormones remodel the microbiota, microbial outputs skew neurotransmitters (low GABA, low 5-HT, high Kyn/Trp, high Glu/NMDA), and these shifts intensify anxiety and depressive symptoms, highlighting neurotransmitter modulation as the proximate pathway through which the microbiota shapes affect. Thus, microbial control of inhibitory (GABA), monoaminergic (5-HT/DA/NE), and glutamatergic (NMDA) systems provides a direct, mechanistic bridge from gut dysbiosis to the core symptom dimensions of anxiety and depression, and a tractable target for biomarker-guided interventions (60, 83–85).

## Microbiota–endocrine crosstalk and hpa axis dysregulation

The HPA axis is the central coordinator of the stress response, and its dysregulation is strongly implicated in anxiety and depression (86). Emerging evidence indicates that the gut microbiota critically shapes HPA axis activity through endocrine and metabolic pathways, creating a reciprocal loop in which microbial communities influence stress hormones while glucocorticoids remodel the gut ecosystem (87, 88).

Preclinical studies provide direct behavioral evidence that microbiota–endocrine interactions modulate anxiety- and depression-like outcomes. In rodent models, adolescent stress not only induces hippocampal BDNF downregulation and cognitive impairments but also produces pronounced anxiety- and depression-like behaviors, such as increased immobility in the forced swim test (FST), reduced sucrose preference (SPT), and enhanced avoidance in the elevated plus maze (EPM), changes that parallel gut microbial dysbiosis (89, 90). Dietary interventions with omega-3 polyunsaturated fatty acids or vitamin A attenuate these affective phenotypes, restoring exploratory behavior and anhedonia scores, normalizing hippocampal BDNF expression, and partially reestablishing microbial composition, suggesting that microbial–endocrine signaling buffers against HPA hyperactivation (63, 90). Similarly, administration of *Mycobacterium vaccae* confers robust anxiolytic and antidepressant-like effects, reducing immobility in FST, improving open-field exploration, and normalizing corticosterone levels, through Treg-mediated immunomodulation and glucocorticoid dampening, thereby enhancing resilience to chronic stress and inflammation (91).

Clinical findings parallel these experimental observations. Patients with depression and anxiety frequently show altered gut microbial signatures associated with hypercortisolemia and disrupted diurnal cortisol rhythms (92, 93). Translational models confirm that microbiota perturbations heighten HPA axis reactivity and aggravate depressive phenotypes (6).

Importantly, probiotic supplementation with *Bifidobacterium* and *Lactobacillus* strains reduces cortisol levels and improves both mood and stress responsiveness (94, 95). Randomized controlled trials further demonstrate that specific probiotic formulations, such as *Lactobacillus casei* Shirota, *Lactobacillus gasseri* CP2305, and *Bifidobacterium longum* 1714, lower salivary cortisol and attenuate anxiety or stress-induced physiological responses in humans (96–98). Additionally, a multi-strain combination of *L. helveticus* R0052 and *B. longum* R0175 has been shown to reduce urinary cortisol and psychological distress in both preclinical and clinical models (99, 100).

The mechanistic basis of this crosstalk lies partly in microbial metabolites. SCFAs modulate glucocorticoid receptor sensitivity and neurotransmitter release, directly linking bacterial fermentation products to stress hormone regulation (101). Developmental studies add another layer of complexity: germ-free animals display exaggerated ACTH and corticosterone responses to stress, which normalize only when colonization with *Bifidobacterium infantis* occurs during early life, revealing a sensitive period for microbiota-driven endocrine programming (102). In humans, early-life adversity correlates with persistent microbial alterations and long-term cortisol dysregulation, suggesting that developmental disruptions in microbiota–endocrine communication may predispose individuals to psychiatric vulnerability (90). These developmental findings emphasize that timing of microbial exposure, particularly during critical windows such as infancy and adolescence, can have enduring consequences on HPA-axis regulation and stress resilience.

Notably, the HPA axis is tightly regulated by circadian clocks. Dysbiosis-driven cortisol abnormalities often coincide with flattened diurnal rhythms, reinforcing maladaptive stress responses and increasing susceptibility to anxiety and depression (103–105). Circadian misalignment not only alters cortisol rhythmicity but also disrupts gut microbial oscillations, amplifying systemic inflammation and stress sensitivity. Chronotherapeutic interventions that restore circadian–microbial synchrony, such as time-restricted feeding, chrononutrition, and light–sleep alignment, are now being investigated as strategies to recalibrate endocrine and microbial rhythms in mood disorders (31, 44, 106). Together, these findings establish the microbiota as an active regulator of HPA axis function. By integrating endocrine, immune, and circadian control, microbial communities determine the threshold and recovery of stress responses. Dysbiosis amplifies stress hormone release and psychiatric vulnerability, whereas targeted interventions, including psychobiotics, prebiotics, dietary supplementation, and timing-aligned (chronobiotic) interventions, offer promising avenues to restore endocrine balance and enhance resilience against anxiety- and depression-related disorders (107).

## Neuroimmune mechanisms linking gut dysbiosis to anxiety and depression

Gut dysbiosis profoundly influences the immune system, triggering a cascade of neuroimmune alterations that extend to the CNS (108, 109). Perturbations in microbial composition promote chronic low-grade inflammation through the release of pathogen-associated molecular patterns (PAMPs) and danger-associated molecular patterns (DAMPs), which engage pattern recognition receptors such as Toll-like receptors and NOD-like receptors (110). These signals activate inflammasomes and upregulate proinflammatory cytokines, including IL-1β, IL-6, and TNF-α, which in turn modulate microglial activity and neural plasticity, fostering vulnerability to psychiatric disorders (110–112). Seminal frameworks now link sustained peripheral inflammation to “sickness behavior” and depression *via* immune-to-brain signaling, anchoring dysbiosis-driven inflammation within modern depression pathophysiology (15).

A study demonstrates that gut microbiota disturbances compromise the BBB, facilitating peripheral immune mediators’ access to the brain (113). Chronic social stress reduces tight-junction protein claudin-5, breaches the BBB, and enables peripheral IL-6 infiltration into limbic circuits, driving depression-like behaviors; convergent work confirms stress-induced BBB pathology across models (114). Beyond animal models, vascular activation markers, including soluble adhesion molecules, are altered in depression, and in women with MDD, circulating sE-selectin is significantly elevated, underscoring sex-linked endothelial contributions to neuroimmune dysregulation (115). Cytokines can also access or signal across the BBB *via* endothelial and perivascular routes, providing direct channels by which peripheral immune tone shapes CNS function (116).

At the cellular level, gut-derived metabolites further orchestrate neuroimmune interactions. SCFAs, normally protective, become depleted in dysbiosis, removing a key brake on histone deacetylases and G-protein-coupled receptor signaling (117, 118). This loss enhances microglial reactivity and reduces regulatory T cell function, driving maladaptive inflammatory signaling (17, 119). Additionally, kynurenine pathway metabolites, shaped by microbial metabolism of tryptophan, engage the AhR to promote Treg expansion and dampen cytotoxic T cell activity, thereby linking metabolic disturbances to immune escape and neurodegeneration (120–123). Microglial NLRP3 inflammasome activation has emerged as a central node that converts stress and metabolic danger signals into IL-1β-driven neuroinflammation and depressive-like behavior, highlighting druggable checkpoints within the gut–immune–brain axis (124, 125).

Sterile inflammation, independent of pathogens, can be initiated by psychological stress and gut dysbiosis, culminating in increased NLRP3 activation and IL-1β release in both the periphery and the CNS (125–127). These processes compromise neuronal survival and synaptic plasticity, fostering cognitive and mood disturbances. Furthermore, peripheral immune activation translates into CNS changes *via* vagal and humoral routes, with immune-to-brain signaling shaping behavioral responses to stress and contributing to depression and anxiety phenotypes (128). Importantly, human molecular-imaging studies show increased brain TSPO binding, a proxy for microglial activation, during major depressive episodes, providing *in vivo* evidence that heightened neuroinflammation accompanies symptom severity (129).

Microglia, the resident immune cells of the brain, are especially sensitive to gut microbial signals. Dysbiosis-induced priming of microglia amplifies proinflammatory cytokine release and impairs phagocytic clearance of pathological proteins. This dysfunctional state is closely linked to aberrant synaptic pruning and neuronal circuit remodeling (3). Crucially, systemic immune alterations dictate microglial phenotypes: chronic elevations of IL-6, TNF-α, and IL-1β “prime” microglia, lowering activation thresholds and exaggerating responses to subsequent stressors, whereas IL-10 and TGF-β bias microglia toward homeostatic states. This systemic-to-central coupling reframes microglia as sensors of peripheral immune tone, not merely local responders (3). Microbiota are indispensable for normal microglial maturation and function, and their depletion disrupts microglial transcriptional homeostasis, further connecting gut ecology to central immune set-points (130). Parallel evidence shows that immune-cell trafficking into the CNS, facilitated by BBB disruption and chemokine gradients, intensifies neuroinflammation and exacerbates behavioral deficits (114, 131, 132).

Clinical and translational evidence corroborates these mechanistic insights. Patients with depression exhibit systemic inflammation, BBB leakage, and altered immune signatures in brain tissue, providing convergent evidence that gut dysbiosis-driven neuroimmune dysregulation is not a secondary phenomenon but a mechanistic driver of psychiatric pathology (133, 134). Finally, circadian control powerfully shapes these processes: immune transcripts and cytokines oscillate across the day, and circadian misalignment amplifies pro-inflammatory output and microglial reactivity, linking disrupted temporal organization to persistent neuroinflammation in mood disorders (135–138).

## Metabolic mediators of microbiota–brain communication

The gut microbiota generates a wide array of metabolites that directly and indirectly influence brain physiology, many of which have been implicated in the pathophysiology of anxiety and depression (139, 140). Among the most studied are SCFAs such as acetate, propionate, and butyrate. These molecules act as epigenetic regulators by inhibiting histone deacetylases, thereby modulating the transcription of genes involved in neuroplasticity and stress responses (130, 141, 142). In parallel, SCFAs interact with G protein–coupled receptors, including GPR41 and GPR43, shaping systemic immune activity and favoring the production of anti-inflammatory cytokines, which in turn influence mood regulation (11, 143). Evidence from animal models shows that depletion of SCFA-producing bacteria heightens anxiety-like behaviors, while butyrate supplementation restores synaptic integrity and normalizes HPA axis function (29, 144).

Tryptophan metabolism constitutes another crucial microbiota-controlled pathway linking the gut and brain (18). Beneficial microbes such as *Bifidobacterium* and *Lactobacillus* spp. enhance serotonin biosynthesis by stimulating enterochromaffin cells, influencing central serotonergic signaling through vagal and circulatory routes (18, 145). However, in the context of dysbiosis, tryptophan is preferentially metabolized *via* the kynurenine pathway through activation of IDO, leading to the accumulation of neuroactive metabolites such as quinolinic acid, which acts as an NMDA receptor agonist and exerts neurotoxic effects, and kynurenic acid, which functions as an NMDA antagonist with neuroprotective properties (11, 146). This imbalance has been linked to excitotoxicity, chronic neuroinflammation, and depressive symptomatology, and clinical studies consistently report altered kynurenine-to-tryptophan ratios in patients with MDD (146).

Beyond these classical metabolites, bile acids have recently emerged as key modulators of microbiota–brain communication. Primary bile acids synthesized in the liver undergo microbial transformation into secondary bile acids such as deoxycholic acid and lithocholic acid, which can cross the BBB and act on receptors including FXR and TGR5 (147, 148). These receptors influence systemic metabolism, immune signaling, and microglial activation, processes that collectively shape neural plasticity and emotional regulation. Dysregulated bile acid pools have been associated with impaired hippocampal function, altered energy balance, and enhanced anxiety-like behavior, positioning bile acid signaling as an additional therapeutic target in mood disorders (149, 150). Microbial catabolism of tryptophan also yields indole derivatives such as indole-3-acetic acid and indole-3-aldehyde, which activate the AhR in epithelial and immune cells (151). Activation of this receptor supports intestinal barrier integrity and restrains pro-inflammatory cascades that otherwise propagate systemic inflammation and brain dysfunction. Reduced levels of indole-producing microbes are frequently observed in anxiety and depression, suggesting that impaired indole–AhR signaling contributes to disease pathology and may be restored through microbiome-targeted interventions (151). Lactate, another microbial metabolite, is increasingly recognized not only as a byproduct of glycolysis but also as a key neuroenergetic shuttle. Microbiota-derived lactate fuels astrocyte–neuron metabolic coupling, thereby sustaining synaptic plasticity and modulating neuronal excitability (152). Animal studies indicate that higher microbial lactate production correlates with resilience to stress-induced behavioral alterations, underscoring its neuroprotective role (153). Many of these metabolites, including SCFAs, bile acids, and kynurenine pathway intermediates, exhibit circadian oscillations synchronized with host feeding–fasting cycles. Disruption of these rhythms contributes to metabolic misalignment, impaired neurotransmission, and mood dysregulation (154, 155).

Taken together, these metabolites constitute an interconnected signaling network that links gut microbial activity to brain function. Anxiety and depression are consistently associated with a metabolic signature characterized by reduced SCFAs and indoles, elevated kynurenine pathway metabolites, and disrupted bile acid profiles (142, 146, 150, 156). Therapeutic approaches aimed at restoring microbial balance, through prebiotics, probiotics, or dietary strategies, have demonstrated the capacity to reestablish metabolite production, normalize neurotransmission and inflammatory tone, and ultimately alleviate mood-related symptoms (5).

## Circadian rhythm disruption and the gut–brain axis

Circadian rhythms, generated by transcription–translation feedback loops of core clock genes such as *CLOCK*, *BMAL1*, *PER*, and *CRY*, orchestrate nearly all aspects of physiology, including sleep–wake cycles, hormone secretion, metabolism, and immune responses (157, 158). While the suprachiasmatic nucleus (SCN) serves as the central pacemaker, peripheral clocks in the gut, liver, and immune system also display robust oscillations that synchronize host metabolism and stress responsivity to environmental cues (157). Importantly, the gut microbiota itself undergoes pronounced diurnal oscillations in composition, localization, and metabolite production, which are tightly entrained by host feeding–fasting cycles and circadian signaling (159, 160).

Seminal studies demonstrated that intestinal bacteria coordinate with the circadian system to shape host gene expression and metabolic rhythms. For instance, diurnal microbial relocation along the gut epithelium and rhythmic metabolite fluxes program oscillations in the host liver transcriptome and epigenetic landscape (160). Similarly, the microbiota programs HDAC3-dependent diurnal transcription in the intestine, controlling lipid uptake and energy balance (44). Perturbation of these rhythms, either by antibiotics, germ-free conditions, or arrhythmic feeding, disrupts circadian gene expression and increases susceptibility to obesity and inflammation (50, 161).

Circadian disruption also alters gut–immune interactions. Segmented filamentous bacteria (SFB) exhibit rhythmic attachment to epithelial cells, driving ILC3–STAT3–mediated (80) antimicrobial protein oscillations that enhance time-of-day–specific resistance to infection. These microbiota–immune interactions are synchronized with feeding rhythms, underscoring the integration of diet, circadian clocks, and microbial dynamics. In parallel, the microbiota regulates diurnal corticosterone secretion, modulating HPA axis activity and stress responsiveness (81). Microbial depletion abolishes rhythmicity of corticosterone and stress pathway gene expression in the hippocampus and amygdala, leading to time-of-day–specific impairments in stress resilience (81, 162).

Disruption of circadian rhythmicity, through shift work, jet lag, high-fat diets, or sleep disturbance, induces microbial dysbiosis, dampens metabolite oscillations, and desynchronizes host clocks (50, 158, 161). Human studies show that such misalignment exacerbates anxiety and depression, as circadian disruption not only destabilizes sleep but also amplifies neuroinflammatory signaling and HPA hyperactivity (35). This bidirectional dysfunction creates a feed-forward loop in which stress and microbial imbalance reinforce circadian misalignment, heightening psychiatric vulnerability (81, 160). Integrating circadian biology into the gut–brain research opens promising therapeutic avenues. Chrononutrition and time-restricted feeding restore microbial rhythmicity and improve host metabolic and mood outcomes (50, 159). Likewise, probiotics such as Lactobacillus reuteri have been shown to normalize glucocorticoid rhythms and stress responsivity (81). Emerging chronotherapeutic strategies suggest that aligning microbial interventions such as psychobiotics, FMT, or engineered microbiomes with circadian windows may maximize efficacy for anxiety and depression (35, 157, 160).

## Microbial signatures and biomarkers

The gut microbiome has emerged as a central determinant of neuropsychiatric health, with specific microbial taxa and their metabolites increasingly recognized as candidate biomarkers for anxiety and depression (163). Multi-omics studies demonstrate that microbial dysbiosis directly correlates with immune activation, host metabolism, and brain structure. For example, clinical cohorts revealed altered abundance of *Ruminococcus bromii*, *Lactococcus chungangensis*, and *Streptococcus gallolyticus* in individuals with MDD, with these taxa increasing pro-inflammatory signaling *via* LPS- and peptidoglycan-mediated TLR/NOD pathways, elevating IL-1β and perturbing lipid oxidation, and reductions in grey matter volume in the inferior frontal gyrus (164). Notably, these microbial shifts were associated with functional brain imaging readouts, including altered fronto-limbic connectivity that tracks peripheral cytokine load, providing neuroanatomical correlates of dysbiosis. Extending this, large population-level data from the Flemish Gut Flora Project identified butyrate-producing *Faecalibacterium* and *Coprococcus* as positive correlates of quality of life and inverse correlates of depression scores, where butyrate inhibits HDACs, activates FFAR2/FFAR3, tightens epithelial junctions, and suppresses IL-6/TNF-α, establishing these genera as reproducible, population-scale candidates for biomarker panels (165).

Meta-analyses reinforce these findings by showing consistent depletion of butyrate-producing genera such as *Butyricicoccus*, *Coprococcus*, and *Faecalibacterium*, alongside enrichment of pro-inflammatory taxa including *Eggerthella*, *Enterococcus*, *Flavonifractor*, and *Streptococcus* in depressive patients (41, 166). Loss of SCFA producers reduces mucosal butyrate, weakens claudin/occludin expression, increases microbial translocation, and amplifies NLRP3-IL-1β signaling, both of which are critical drivers of HPA axis overactivation and mood disturbances (167). Such immune-related microbial signatures are increasingly proposed as stratification tools to identify patients likely to benefit from anti-inflammatory or microbiota-directed interventions. Recent systematic syntheses confirm these compositional trends across studies and tie them to quantitative biomarker thresholds (e.g., low fecal butyrate, high kynurenine/tryptophan ratio, elevated LBP/CRP), supporting their use as putative diagnostic and prognostic markers rather than mere correlates (168).

Specific taxa repeatedly appear as microbial signatures of psychiatric dysfunction. *Bifidobacterium* and *Lactobacillus* are frequently diminished in major depression, which reduces microbial GABA production and attenuates enterochromaffin 5-HT biosynthesis *via* TPH1 stimulation, thereby linking gut dysbiosis to neurotransmitter imbalance (11, 169). Conversely, expansion of *Actinobacteria* and *Proteobacteria* has been correlated with inflammatory cytokine release and oxidative stress pathways (170, 171), with Proteobacteria enriching endotoxin load that activates TLR4/NF-κB and elevates IL-6/TNF-α. Importantly, these compositional imbalances have been shown to normalize following probiotic supplementation or targeted diets, accompanied by restored fecal SCFAs, lower Kyn/Trp ratios, and reduced circulating sICAM-1/sVCAM-1, underscoring their translational potential. Causality is supported by FMT: transfer of “depression microbiota” increases hippocampal quinolinic acid, decreases butyrate, and induces microglial priming and depressive-like behavior, whereas healthy-donor microbiota does not, functionally validating these signatures (169).

Integrative omics has further advanced biomarker discovery. Metagenomic and metabolomic studies highlight disrupted tryptophan metabolism as a central pathway, with microbial activation of the kynurenine pathway *via* IDO/TDO diverting tryptophan from 5-HT, accumulating neuroactive kynurenines (quinolinic > kynurenic), and potentiating NMDA-dependent excitotoxicity (164, 165). At the same time, SCFA depletion and altered phosphoethanolamine levels suggest lipid and energy metabolism as crucial biomarker domains for depression and anxiety (165, 168). Across psychiatric cohorts, elevated kynurenine-to-tryptophan ratios and shifts in neuroactive kynurenines (e.g., quinolinic vs. kynurenic acid) are among the most consistent metabolic readouts, and are mechanistically tied to glutamatergic signaling and neuroinflammation (84). Additional axes include bile-acid remodeling, microbial deoxycholic/lithocholic acids that activate FXR/TGR5 and modulate microglial tone, and indole-AhR signaling, where reduced indole-3 derivatives diminish barrier and permit pro-inflammatory drift (172).

Collectively, these findings define an evidence-based biomarker panel that includes butyrate-producing genera such as *Faecalibacterium* and *Coprococcus*, consistently associated with improved quality of life and reduced depressive symptoms (11); depletion of *Bifidobacterium* and *Lactobacillus*, which are linked to diminished production of GABA and serotonin precursors and thus to neurotransmitter imbalance; and enrichment of pro-inflammatory taxa including *Eggerthella* and Enterobacteriaceae, which correlate with heightened cytokine release and neuroinflammation. Metabolically, reduced levels of SCFAs and indole derivatives together with elevated kynurenine-pathway intermediates, long implicated in glutamatergic dysregulation and neurotoxicity, represent reproducible molecular signatures across psychiatric cohorts. Importantly, these microbial and metabolic profiles correlate with clinical outcomes and can transfer depressive phenotypes in FMT models, providing causal validation. Beyond these canonical markers, recent studies highlight bile-acid remodeling and microbial-derived lactate as novel metabolic signatures influencing limbic activity and resilience to stress. Finally, because microbial taxa and metabolites exhibit diurnal oscillations entrained by host circadian rhythms, biomarker sampling and interpretation must account for temporal dynamics, as circadian misalignment can confound associations and obscure their diagnostic and prognostic value (35).

Taken together, these biomarkers not only delineate mechanistic pathways but also serve as direct entry points for therapeutic targeting, bridging discovery with intervention.

## Therapeutic strategies targeting the gut–brain axis in anxiety and depression

The gut–brain axis is increasingly recognized as a modifiable therapeutic target in anxiety and depression, with interventions spanning diet, microbial modulation, targeted microbiota transfers, and host-directed anti-inflammatory strategies (173). Collectively, these approaches demonstrate that reshaping microbiota–brain communication can produce measurable effects on emotional, cognitive, and systemic outcomes. Importantly, such interventions align with the microbial, metabolic, and immune biomarkers described above, offering a foundation for precision psychiatry (**Table 1**).

Among the available strategies, dietary modification remains the most robustly supported intervention. In the landmark SMILES randomized controlled trial, adjunctive dietary support led to a marked reduction in clinician-rated depression (MADRS; Cohen’s d ≈ −1.16) and quadrupled remission compared with an active social-support control (32.3% vs 8.0%) (174). A meta-analysis of 16 randomized clinical trials (RCTs) further confirmed a small-to-moderate pooled benefit of dietary interventions for depressive symptoms (g≈0.28) but not for anxiety, underscoring phenotype-specific effects (175). Long-term prospective evidence from 180,446 UK Biobank participants demonstrated that greater adherence to the EAT-Lancet dietary pattern significantly reduced the risk of depression and anxiety (HR for depression 0.71–0.84) (176). Mechanistic studies reveal that such dietary patterns enrich butyrate-producing taxa, including *Faecalibacterium* and *Coprococcus*, thereby restoring SCFA-mediated signaling and countering the microbiome deficits observed in depressive disorders.

Probiotic and prebiotic supplementation, collectively referred to as psychobiotics, has emerged as a promising adjunctive approach rather than a stand-alone therapy. A comprehensive meta-analysis showed that probiotics significantly reduced depressive symptoms when co-administered with antidepressants (SMD = 0.83), whereas no significant effect was observed in isolation (177). Mechanistic RCTs provide convergent evidence: in MDD, a four-week course of multi-strain probiotics added to standard therapy reduced Hamilton Depression Rating (HAM-D) scores more than placebo, preserved microbial diversity, increased *Lactobacillus* abundance correlated with symptom improvement, and attenuated putaminal reactivity during emotion-processing tasks (178). Similarly, *Bifidobacterium longum* NCC3001 improved depressive symptoms in patients with irritable bowel syndrome and reduced amygdala and fronto-limbic reactivity to negative stimuli, linking peripheral microbial modulation to central neural changes (179). Collectively, these studies support the capacity of psychobiotics to recalibrate neuroimmune and neuroendocrine pathways that underpin mood regulation (180).

FMT is transitioning from proof-of-concept to early clinical application. Preclinical studies demonstrate that transplantation of “depression microbiota” from affected individuals to rodents induces depressive-like behavior, establishing causality between dysbiosis and mood pathology (181). The first double-blind pilot RCT in adults with moderate-to-severe MDD confirmed the feasibility and safety of FMT *via* enema, reporting improved gastrointestinal symptoms and quality of life with no major adverse events (181). Future studies are moving toward precision-matched donor–recipient approaches based on specific biomarker signatures, such as low SCFA or indole levels and elevated kynurenine pathway activity, thereby translating mechanistic findings into personalized interventions.

Host-targeted anti-inflammatory strategies are gaining attention, particularly for inflammation-driven subtypes of depression. In a pivotal trial in treatment-resistant MDD, the TNF-α antagonist infliximab produced no overall advantage over placebo. However, patients with elevated baseline hs-CRP (> 5 mg/L) exhibited significantly greater reductions in HAM-D scores and higher response rates, while those with low CRP worsened on treatment, highlighting the potential harm of non-stratified immunotherapy (182). These results emphasize the necessity of integrating inflammatory biomarkers into treatment algorithms and reserving biologic agents for well-defined, inflammation-positive subgroups.

A rapidly advancing frontier integrates circadian biology into gut–brain therapeutics, aligning microbial and host rhythmicity to optimize efficacy. Chrononutrition and time-restricted eating (TRE) restore microbial diurnal oscillations, re-establish SCFA rhythmicity, and improve metabolic and mood-related parameters. Although meta-analyses report variable outcomes, consistent early-day feeding schedules (eTRE; 8 a.m.–4 p.m.) have been shown to enhance circadian synchrony and lower stress reactivity (155, 183–185). In parallel, chrono-probiotics, specifically *Lactobacillus gasseri* CP2305, exhibit stress-buffering and sleep-regulating properties, reducing salivary cortisol and improving subjective sleep quality in chronically stressed adults (186, 187). These findings indicate that synchronizing probiotic administration with host circadian cycles may potentiate neuroendocrine resilience and mood stability.

Beyond microbial interventions, circadian behavioral therapies are now a core component of integrated treatment frameworks. Morning bright-light therapy (BLT) acts as a circadian phase-advancing intervention that accelerates antidepressant response and improves outcomes in nonseasonal depression, with meta-analyses confirming enhanced remission and response rates when combined with pharmacotherapy or cognitive behavioral therapy (Lieverse et al., 2022; Legenbauer et al., 2024; Perera et al., 2016). These interventions normalize melatonin and cortisol rhythmicity, thereby complementing microbiota- and metabolism-targeted strategies.

When these findings are considered together, a coherent therapeutic hierarchy emerges. Dietary modification represents a scalable, evidence-based foundation for depression prevention and treatment, while anxiety may require more targeted or multimodal approaches (174–176). Psychobiotics serve as effective adjuncts that modulate neurocircuitry and immune tone (177, 178). FMT remains investigational but increasingly feasible, emphasizing donor selection and mechanistic alignment (179, 181). Inflammation-stratified biologics may benefit well-defined inflammatory subgroups (182). Finally, circadian-aligned strategies, chrononutrition, chrono-probiotics, and BLT, extend these interventions into the temporal dimension, restoring synchronization across the gut–brain–immune axis. Across modalities, precision gut microbiota modulators, encompassing diet, probiotics, engineered consortia, bacteriophages, and targeted delivery systems, should be conceptualized as biomarker- and rhythm-guided tools rather than one-size-fits-all therapies (188).

Despite these advances, important limitations persist. Probiotic and FMT studies remain small, heterogeneous in strains, dosing, and duration, and often confounded by comorbidities and concomitant medications (178, 179). Dietary interventions consistently demonstrate antidepressant efficacy but less impact on anxiety (175), and anti-TNF strategies illustrate the risks of non-stratified application (182). These limitations reinforce a stepped, integrated model: optimize diet as the foundational layer; incorporate psychobiotics with biomarker and behavioral monitoring; introduce circadian-aligned interventions such as light therapy, regular feeding rhythms, and sleep regulation; and reserve immunomodulation for biomarker-defined inflammatory phenotypes. Altogether, these therapeutic layers anchor treatment to the molecular, microbial, and temporal signatures of psychiatric disease, defining a roadmap for biomarker- and chronobiology-informed precision psychiatry (189).

## Conclusions and future perspectives

The collective evidence presented across current studies underscores that the gut–brain–circadian axis is a central regulatory hub for mental health, particularly in the context of anxiety and depression (190, 191). Mechanistic insights consistently demonstrate that microbial metabolites such as SCFAs, bile acids, and tryptophan derivatives influence neuronal signaling, immune activity, and endocrine homeostasis, ultimately shaping stress responses and emotional regulation (4, 6, 11). Dysbiosis-driven alterations in vagal, HPA-axis, and immune pathways provide a causal basis for mood-related disorders, while circadian disruption emerges as a critical modifier that amplifies these effects by desynchronizing host–microbiome rhythms (15, 36). Together, these findings highlight the necessity of considering circadian alignment as an integral component in gut–brain research and therapy design.

Therapeutically, the data indicate that multiple strategies hold promise in modulating the gut–brain axis. Diet remains the most clinically validated intervention, with trials demonstrating robust improvements in depression following structured dietary modifications (3). Beyond diet, probiotics, prebiotics, and psychobiotics target microbial communities with the potential to restore balanced signaling through neuroimmune and neuroendocrine pathways (4). More advanced modalities such as FMT, engineered microbial consortia, and bacteriophage-based interventions are gaining attention, though their long-term efficacy and safety require further validation (14). Anti-inflammatory and immunomodulatory strategies targeting microbiota-driven neuroinflammation also show preclinical efficacy, opening an avenue for adjunctive therapies in patients with treatment-resistant depression and anxiety (3).

A critical frontier lies in multi-omic integration and systems biology approaches. Current multi-omic platforms have begun to delineate disease-associated microbial modules that link species, pathways, and metabolites into coherent signatures predictive of psychiatric outcomes (9, 192). However, challenges persist in harmonizing complex datasets across cohorts, accounting for inter-individual variability, and defining causality rather than correlation. Artificial intelligence and machine learning pipelines, applied to large-scale, longitudinal microbiome and host-omic datasets, will be essential to establish predictive biomarkers and patient-tailored interventions (7, 193).

Importantly, developmental and aging trajectories define sensitive “age windows” during which microbiota–brain–circadian interactions exert disproportionate influence on psychiatric vulnerability. Early-life stages such as the perinatal period and adolescence represent critical windows for microbial and circadian programming of the HPA axis and neuroimmune circuits, with disruptions during these phases leading to long-term effects on stress responsivity and mood regulation (194, 195). In contrast, midlife transitions and aging are characterized by inflammaging, reduced microbial diversity, and dampened circadian amplitude, processes that compromise resilience and increase vulnerability to anxiety and depression (19, 196). These life-stage–specific vulnerabilities also open therapeutic opportunities: early interventions with breastfeeding, structured diet, and chrononutrition may prevent maladaptive trajectories, while targeted psychobiotics, dietary polyphenols, and circadian-aligned sleep–light hygiene may restore resilience in midlife and older age. Thus, “when” to intervene becomes as critical as “what” intervention is applied.

Looking forward, three research avenues appear most critical. First, precision psychiatry must integrate circadian biology, microbial ecology, and life-stage–specific vulnerabilities with host genetics to optimize interventions for individual patients (197). Second, longitudinal and life-course studies are required to capture the developmental windows during which microbiota–brain interactions exert the strongest influence, particularly in childhood, adolescence, and aging (6). Third, clinical translation should focus on designing controlled, reproducible interventions that bridge dietary, microbial, and pharmacological approaches, while monitoring adverse outcomes such as immune-related side effects or unintended microbial shifts (36). In conclusion, the gut–brain–circadian framework offers a transformative paradigm for understanding and managing anxiety and depression. By advancing mechanistic dissection, leveraging integrative omics, and prioritizing translational research, this field is poised to deliver personalized, biologically grounded interventions that could reshape psychiatric care in the coming decade.
